# Potential of ESBL-producing *Escherichia coli* selection in bovine feces after intramammary administration of first generation cephalosporins using in vitro experiments

**DOI:** 10.1038/s41598-022-15558-z

**Published:** 2022-09-05

**Authors:** David C. Speksnijder, Nonke E. M. Hopman, Nina E. Kusters, Arjen Timmerman, Jantijn M. Swinkels, Pleun A. A. Penterman, Volker Krömker, Andrew J. Bradley, Nadine Botteldoorn, Ronette Gehring, Aldert L. Zomer

**Affiliations:** 1 Division Infectious Diseases & Immunology, Department of Biomolecular Health Sciences, Faculty of Veterinary Medicine, Utrecht University, Utrecht, The Netherlands; 2 University Farm Animal Clinic, Harmelen, The Netherlands; 3 Global Ruminant Business Unit, MSD Animal Health, Boxmeer, The Netherlands; 4 MSD Animal Health, Boxmeer, The Netherlands; 5 Department of Veterinary and Animal Sciences, University of Copenhagen, Copenhagen, Denmark; 6 Quality Milk Management Services Ltd, Cedar Barn, Easton, Wells UK; 7 School of Veterinary Medicine and Science, Sutton Bonington Campus, University of Nottingham, Sutton Bonington, Leicestershire, LE12 5RD UK; 8 Animal Health Care Flanders, Torhout, Belgium; 9 Department of Population Health Sciences, Faculty of Veterinary Medicine, Institute for Risk Assessment Sciences, Utrecht University, Utrecht, The Netherlands

**Keywords:** Pathogens, Antibiotics, Antimicrobial resistance, Bioinformatics, Public health

## Abstract

Selection and spread of Extended Spectrum Beta-Lactamase (ESBL) -producing *Enterobacteriaceae* within animal production systems and potential spillover to humans is a major concern. Intramammary treatment of dairy cows with first-generation cephalosporins is a common practice and potentially selects for ESBL-producing *Enterobacteriaceae*, although it is unknown whether this really occurs in the bovine fecal environment. We aimed to study the potential effects of intramammary application of cephapirin (CP) and cefalonium (CL) to select for ESBL-producing *Escherichia coli* in the intestinal content of treated dairy cows and in manure slurry, using in vitro competition experiments with ESBL and non-ESBL *E. coli* isolates. No selection of ESBL-producing *E. coli* was observed at or below concentrations of 0.8 µg/ml and 4.0 µg/ml in bovine feces for CP and CL, respectively, and at or below 8.0 µg/ml and 4.0 µg/ml, respectively, in manure slurry. We calculated that the maximum concentration of CP and CL after intramammary treatment with commercial products will not exceed 0.29 µg/ml in feces and 0.03 µg/ml in manure slurry. Therefore, the results of this study did not find evidence supporting the selection of ESBL-producing *E. coli* in bovine feces or in manure slurry after intramammary use of commercial CP or CL-containing products.

## Introduction

Given the worldwide increase of antimicrobial resistance (AMR), efforts have been made in both human and veterinary medicine to prevent AMR selection and spread^1–4^. From a One Health perspective, the selection and spread of Extended Spectrum Beta-Lactamase (ESBL)-producing *Enterobacteriaceae* (ESBL-pE) within animal production systems and potential spillover to humans is a major concern^5–8^. When trying to curb AMR, it is important to limit the spread of infections on one hand, and to eliminate inappropriate antimicrobial use (AMU) on the other hand^9^. The use of beta-lactam antibiotics, especially the use of extended-spectrum cephalosporins, in (food producing) animals is regarded as a risk factor for the emergence and spread of ESBL-pE^10^. Therefore, the 1st–2nd and 3rd–5th generation cephalosporins are classified by the World Health Organisation (WHO) as Highly Important and Highest Priority Critically Important Antimicrobials for human medicine respectively. These antimicrobials should accordingly be used rationally and restrictively in veterinary medicine^11^.

The selection and spread of antimicrobial-resistant *Enterobacteriaceae* occurs mainly in the gastrointestinal tract (GIT)^12^. There is clear evidence that the use of extended-spectrum cephalosporins (3rd and 4th generation) is associated with a higher prevalence of ESBL-pE on dairy farms^10,13,14^. However, there is less evidence that the use of 1st and 2nd generation cephalosporins selects for ESBL-pE in the bovine GIT or in manure slurry, which could be a major route for transmission of ESBL-pE.

First-generation cephalosporins like cephapirin (CP) and cefalonium (CL) are extensively used worldwide to treat and prevent intramammary infections in dairy cattle^10,13–17^. This raises the question whether 1st generation cephalosporins applied intramammary can select for ESBL-pE in the GIT of treated dairy cows, or in the environment once they are excreted via feces or urine or discarded via waste milk in the slurry pit. The aim of this study was to gather scientific evidence on the risks for selection and spread of AMR after intramammary treatment of (sub)clinical mastitis in dairy cows with CP and CL and the associated ESBL selection in the bovine gut and in manure slurry, by using in vitro selection experiments.

## Results

### Presumptive concentration of cephapirin and cefalonium in the bovine gut

Taking the found findings from literature, we can calculate the maximum concentration of CP and CL in the gut, where it might select for ESBLs (Table 1A and B). The calculated maximum concentration of CP in feces after intramammary application is 0.114 µg/ml feces, and for CL is 0.289 µg/ml feces.

**Table 1:** Assumed maximum concentration of CP, in the bovine gut after intramammary treatment (IMM) during lactation (A), of CL in the bovine gut after IMM at drying off (B), of CP in manure slurry after IMM during lactation in a 100 cow dairy herd (C), and of CL in manure slurry after IMM at drying off in a 100 cow dairy herd (D).

		Concentration CP/CL per unit	References
**A. Assumptions maximum CP concentration in the gut after intramammary treatment in lactation**			
Administered dose	2 injectors/day with 300 mg CP for 2 consecutive days	600 mg/animal/day	^18^
Percentage systemically absorbed	41%	246 mg/animal/day	^15,19^
Percentage excreted via feces (biliary route)	2%	4.92 mg/animal/day	^18^
Daily feces production adult cow	43.2 kg/day ^1^	0.114 µg/g feces, equivalent to 0.114 µg/ml feces ^2^	^20,21^
**B. Assumptions maximum CL concentration in the gut after intramammary treatment at drying off**			
Administered dose	4 injectors with 250 mg CL, once	1000 mg/animal	^22,23^
Percentage excreted via feces (biliary route)	2% (over a period of 3 days)	6.7 mg/animal/day	
Daily feces production adult cow	23.2 kg/day ^3^	0.289 µg/g feces, equivalent to 0.289 µg/ml feces	^20^
**C. Assumptions maximum CP concentration in manure slurry after intramammary treatment in lactation (100 cow herd)**			
Mastitis treatment incidence	50 treatments/year of 4*300 mg CP	1200 mg CP/treatment	^24^
CP in slurry pit	Assuming all active substance enters the slurry pit via discarded milk, urine and feces	60,000 mg CP/year in slurry pit	
Daily slurry production adult cow	70 kg/day/lactating cow	2,555,000 kg slurry/year	^20^
Maximum concentration CP in slurry		0.023 µg/g slurry, equivalent to 0.023 µg/ml slurry ^4^	
**D. Assumptions maximum CL concentration in manure slurry after drying off (100 cow herd)**			
Dry cow treatment incidence	75 treatments/year of 4*250 mg CL ^5^	1000 mg CL/treatment	^NA^
CL in slurry pit	Assuming all active substance enters the slurry pit via discarded milk, urine and feces	75,000 mg CL/year in slurry pit	^NA^
Daily slurry production adult cow	70 kg/day	2,555,000 kg slurry/year	^20^
Maximum concentration CL in slurry		0.029 µg/g slurry, equivalent to 0.029 µg/ml slurry	

^1^Daily manure production lactating cow 66.3 kg minus 23.1 kg urine production^21^. Low estimation of fecal production per cow per day as found in the literature.

^2^Assumption density of feces 1.0 kg/L.

^3^Daily manure production dry cow 38.6 kg minus 15.4 kg urine production^21^. Low estimation of fecal production per cow per day as found in the literature.

^4^Slurry density 62lbs/ft^3^ = 0.993 kg/L^20^.

^5^Assumption 25% replacement by heifers and calving interval 365 days makes 75 cows that are dried off every year.

### Presumptive concentration of cephapirin and cefalonium in manure slurry

As described above, administered CP and CL are largely excreted via milk and urine. In daily dairy practice, both discarded milk and urine end up in the manure slurry, which is the mixture of feces, urine, discarded milk, cleaning water etc. To calculate the concentration of CP and CL in manure slurry, several assumptions were made (Table 1C and D). The calculated maximum concentration of CP in slurry is 0.023 µg/ml slurry, and for CL is 0.029 µg/ml slurry.

### Selection on cefotaxime resistance

**Figure 1:**
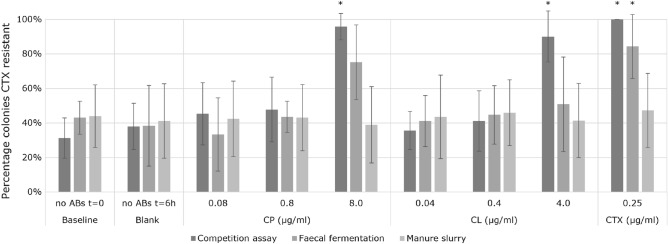
Average and standard deviation of the percentage of CTX resistance of *E. coli* in rich media, fecal fermentation and manure slurry experiments at different concentrations of CP, CL and CTX. * p value ≤ 0.05.

Data on cefotaxime (CTX) resistance (ESBL-producing *Escherichia coli* indicator) in the three sets of experiments (rich media, fecal fermentation, and slurry) and the average for each country are shown in Table 2. Figure 1 shows the average frequency of resistance to CTX in these three sets of experiments. In the rich media experiments, a significant increase in CTX resistance was observed between overnight incubation with 8 µg/ml of CP and 4 µg/ml of CL compared to the blank samples (58% increase, *p* = 0.007 and 52% increase, *p* = 0.023, respectively), using a paired T-test. A significant increase in CTX resistance was also observed in the positive controls (0.25 µg/ml CTX, *p* = 0.003). At lower concentrations no significant increase in CTX resistance was observed. In the fecal fermentation experiments, a borderline significant (*p* = 0.079) increase of 37% in CTX resistance was observed for CP and a non-significant (*p* = 0.190) increase of 13% for CL at the highest concentrations comparing with the blank samples. The positive controls had a significant increase in CTX resistance (*p* = 0.034). For lower concentrations, a very small increase in CTX resistance was observed. In the manure slurry experiments, no increase in CTX resistance or other resistances were observed for any of the applied CP and CL concentrations.

**Table 2:** CTX resistance per different concentration of antimicrobial and per country in the three experiments.

	Baseline	Blank	CP (µg/ml)			CL (µg/ml)			CTX (ug/ml)
	(no ABs t = 0 h)	(no ABs t = 6 h/overnight ^1^)	0.08	0.80	8.00	0.04	0.40	4.00	0.25
**Competition assay in rich media**									
The Netherlands	44%	38%	43%	52%	100%	39%	40%	100%	100%
United Kingdom	17%	20%	21%	24%	100%	25%	21%	100%	100%
Germany	36%	51%	60%	70%	99%	50%	64%	92%	100%
Belgium	28%	44%	57%	45%	84%	29%	41%	69%	100%
Average	31%	38%	45%	48%	96%	36%	41%	90%	100%
st.dev	11.6%	13.4%	18.1%	18.8%	7.7%	11.1%	17.5%	14.8%	0.0%
*p* value	NA	NA	0.073	0.100	**0.007**	0.622	0.416	**0.023**	**0.003**
**Fecal fermentations**									
The Netherlands	51%	45%	63%	56%	96%	45%	57%	76%	92%
United Kingdom	31%	21%	18%	39%	88%	28%	32%	38%	100%
Germany	51%	69%	35%	44%	71%	60%	61%	71%	88%
Belgium	41%	19%	18%	36%	47%	31%	28%	18%	58%
Average	43%	38%	33%	44%	75%	41%	45%	51%	84%
st.dev	9.6%	23.4%	21.2%	9.1%	21.6%	14.8%	16.9%	27.4%	18.5%
*p* value	NA	NA	0.664	0.645	0.079	0.582	0.265	0.190	**0.034**
**Manure slurry**									
The Netherlands	56%	33%	26%	41%	35%	34%	38%	30%	34%
United Kingdom	19%	19%	23%	17%	11%	15%	31%	17%	24%
Germany	43%	70%	69%	58%	65%	71%	74%	61%	66%
Belgium	58%	43%	52%	56%	45%	54%	42%	57%	65%
Average	44%	41%	42%	43%	39%	44%	46%	41%	47%
st.dev	18.2%	21.5%	21.9%	19.1%	22.1%	24.2%	19.0%	21.5%	21.5%
*p* value	NA	NA	0.737	0.746	0.418	0.505	0.177	0.966	0.355

^1^Rich media t = 6 h; fecal fermentations and manure slurry t = overnight; ABs = antibiotics; CP = cephapirin; CL = cefalonium; CTX = cefotaxime.

Significant values are in [bold].

### Metagenomic sequencing

**Figure 2:**
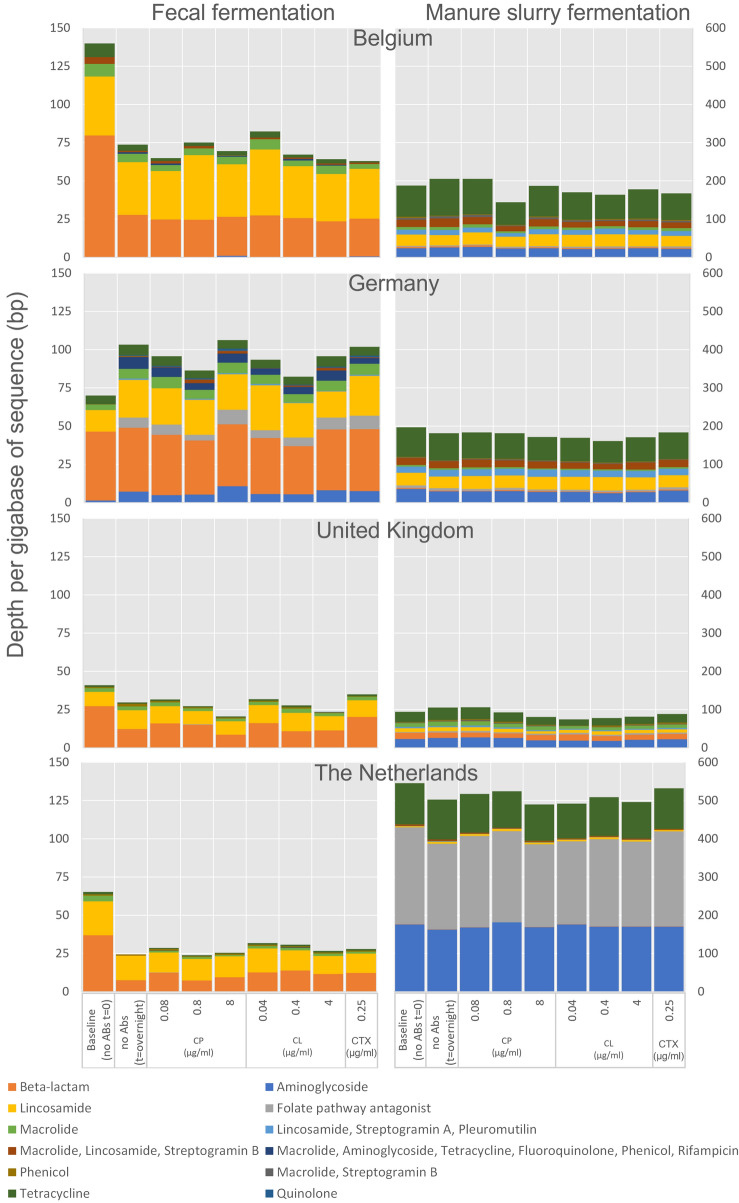
Allignments to resistance genes summed in the fecal fermentations and in manure slurry in the baseline samples, blank samples without antibiotics and per different concentrations of CP, CL and CTX for the four individual countries Belgium (BEL), Germany (GER), the United Kingdom (UK) and the Netherlands (NL).

The resistome was determined for both the fecal fermentation and the manure slurry experiments (Fig. 2), separately for the four countries (Baseline; Blank; 0.08, 0.8 and 8.0 µg/ml CP; 0.04, 0.4 and 4.0 µg/ml CL and 0.25 µg/ml CTX). If we compare all t = 0 values between manure and fecal samples, paired on country, a higher level of reads mapping to resistance genes per gbase is observed in manure slurry however this result is not significant (3.2 fold, *p* = 0.09). Interestingly, we observe higher levels of beta-lactam resistance in the fecal fermentation samples from all countries compared to the manure slurry samples (eightfold difference, *p* = 0.05). In the manure slurry, significantly higher levels of tetracycline resistance where observed (16 fold, *p* = 0.02), and a nonsignificant difference of 210-fold for aminoglycoside resistance (*p* = 0.18). The increased levels of tetracycline resistance vs the decreased levels of beta-lactam resistance are potentially caused by the much higher stability of tetracyclines as compared to beta-lactam antibiotics^25^. Overall, no increase in resistance genes was observed in any of the experiments and using different concentrations of the tested antibiotics.

## Discussion

In our study, we did not observe a significant selection of ESBL-producing *E. coli* at concentrations of ≤ 0.8 µg/ml and ≤ 4.0 µg/ml in bovine feces for CP and CL, respectively, and ≤ 8.0 µg/ml and ≤ 4.0 µg/ml, respectively, in manure slurry. This indicates that the Minimum Selective Concentration (MSC) to select for ESBL-producing *E. coli* in the bovine gut and in manure slurry for CP and CL are ≥ 8.0 µg/ml and 4.0 µg/ml, respectively. Based on our calculations extracted from the available literature, the maximum expected concentrations of CP and CL, after intramammary administrations with doses according to the Summary of Product Characteristics (SPC), will be around 0.11 and 0.29 µg/ml in the feces of adult dairy cows, respectively, and around 0.02 and 0.03 µg/ml in manure slurry, respectively. However, several strengths and weaknesses in our study should be considered.

### Strengths and weaknesses of the study

In the calculations related to the pharmacokinetics of CP and CL and their concentrations in the bovine gut and manure slurry, some general assumptions had to be made. In our assumptions, we took the conservative approach (calculated with the highest possible concentrations of CP and CL in the gut and in manure) to minimize the risk of drawing the wrong conclusion that intramammary applied CP and CL do not select for ESBL-producing *E. coli* in the bovine gut. In regard to the calculations on CL, some issues were ignored as little scientific information exists on its pharmacokinetics which is mostly from small experiments with a limited number of animals^22,23^. We assumed that 41% of the administered dose of CP is absorbed into the circulation as this percentage was the highest found in the literature. Presumably it will be lower, as found in other studies^15,18,19^. As no information is available on the systemic absorption of CL after intramammary treatment, we calculated with 2% fecal excretion of the initial dose in 3 days following treatment according to the summary report provided by EMA^23^. Furthermore, in our calculations, we have not considered the fact that in vivo both CP and CL are quickly degraded by beta-lactamases in the appendix and colon and the metabolism of CP to its less potent desacetyl derivate which already occurs in the mammary gland after administration^26–29^. Additionally, in calculating the average daily fecal production, we have used the lowest fecal production per cow per day found in the literature (respectively 43.2 and 23.2 kg/cow/day for lactating and dry cows), which means that we calculated the highest possible concentrations of CP and CL in the gut content and in manure slurry. Daily fecal production is mainly dependent on feed intake, digestibility, and dry matter (DM) content of the feces. When calculating with a range of daily feed intake of 18–24 kg DM with a digestibility between 60 and 70% and a DM content of 15%, the daily fecal production of lactating dairy cows will range from 40.0 to 64.0 kg/cow/day with an average of 49.0 kg/cow/day (personal communication Dr. Thomas Schonewille, Utrecht University). For dry cows, a recent study found a daily fecal production in dry cows of 52.7 kg/cow/day 4 weeks before calving (personal communication Dr. Thomas Schonewille, Utrecht University)^30^. These aspects might overestimate the calculated concentrations of CP and CL in feces and manure slurry in our study.

On the other hand, in our calculation an ‘average’ daily fecal excretion was assumed, whereas literature shows that peak fecal excretion of CP and CL is within a couple of hours after (intramammary) administration^15,18^. It is therefore probable that the peak concentration of CP and CL in the intestinal content several hours after administration will be higher than the calculated 24 h maximum average concentration of 0.114 µg/ml and 0.289 µg/ml in lactating and dried off cows respectively. However, after intravenous infusion of a dose of over 5000 mg CP in adult dairy cows, peak concentrations in bile of around 10 µg/g have been measured^18^. This implies that the peak concentration in bile after intramammary administration of 300 mg CP will be far below 1.0 µg/g. Bile, and thus CP and CL, will also be diluted in the gut by intestinal contents.

It is important to consider that pharmacokinetics is often evaluated in healthy animals. The health status of an animal can influence both pharmacokinetics and pharmacodynamics due to various factors. In case of (sub)clinical mastitis, as a result of deeper tissue penetration in infected quarters, systemic absorption of the drug will be higher^18,31,32^. This might result in increased excretion of CP or CL in the gut lumen, however, to which extent this occurs is unknown.

As can be seen in Figs. 1 and 2, there were differences in the parameters obtained from the samples of the different countries. As we randomly selected only a few ESBL producing *E. coli* isolates from the different countries, there will inherently be variation in resistance genes of these isolates and subsequently resistance levels. The fact that the (non-)selection of ESBLs at different concentrations of CP and CL is quite consistent across the countries shows the robustness of our findings.

Based on our results from the competition experiments and genomic analyses, it can be concluded that selection for ESBL resistant *E. coli* strains occurs when a combination of three CTX resistant strains and three susceptible strains of *E. coli* are grown in LB medium for six hrs at 37 °C under aerobic conditions, and exposed to increasing concentrations of beta-lactam antimicrobials. Using a similar approach in fresh feces or manure slurry with overnight anaerobic incubation, minor or no selection of *E. coli* strains was observed, indicating that selection does not or only marginally occur at the tested concentrations in these more complex environments (i.e. feces and manure slurry). We hypothesize that the MSC in complex environments is higher or the selection for resistance is reduced. A similar observation was made in porcine fecal communities with different antibiotics (i.e. increasing gradients of gentamcin and kanamycin). Although difference resistance mechanism are at play, the MSC appeared to be increased by more than one order of magnitude for both antibiotics when embedded in the community due to an increase in the cost of resistance and community protection for susceptible phenotypes^33^.

Fecal fermentations have been used before to study effects of various interventions on gut microbiomes and it forms an attractive and cost-effective method without making use of test subjects or animal models^34–40^. The competition assays in our experiments (in rich media, fecal fermentations, and manure slurry) were all performed in well controlled laboratory circumstances opposed to the normal, uncontrolled and complex situation in an animal or in its environment. Besides, samples originated from only a limited number of animals, from a limited number of farms and countries. A complex situation has thus been simplified to well controlled laboratory settings. To be able to extrapolate these data to real farm settings in living animals, more research in vivo is needed. However, in vivo studies are complicated, expensive and require the use of animals which raises ethical concerns.

### Antimicrobial residues in milk as selective pressure for AMR

Milk from dairy cows under antibiotic treatment for mastitis must be retained, following the recommended withholding period. However, this milk is frequently fed to calves, before the due time, posing a risk for AMR selection on calves’ gut, even at very low residue concentrations^41,42^. Additionally, colostrum of freshly calved cows may contain CP or CL residues (used for dry cow therapy), which may affect the newborn calf intestinal flora by imposing a selective pressure for resistant bacteria. Several studies have been conducted to assess the presence of antimicrobial residues in colostrum directly post-partum after CP dry cow therapy. Church et al. (2008), found that in the first milking after calving, 5% of the dairy cows (n = 156) tested positive for CP residues using a commercially available screening test (Delvotest), but the proportion of positive cows rapidly declined after several milkings^43^. However, Hausler et al. (2013), did not find CP residues in milk after 30 days dry period in 12 dairy cows with a detection limit of 4.1 µg/kg^44^. For CL, EMA suggests that maximum CL concentrations in first milking colostrum will be less than 0.5 µg/g in cows with dry periods of less than 17 days. After a dry period of 29 days the concentration of CL in the first milking post-partum will most probably not exceed 0.2 µg/g^22^. In a recent study in the Netherlands, Hordijk et al. (2019), found no difference in ESBL/AmpC shedding between calves fed colostrum from cows with and without antimicrobial dry-off therapy, although it should be stated that cephalosporins are not used as dry cow therapy in the Netherlands since 2014 (in contrast to Germany, UK and Belgium where they are still being used)^5^. These findings indicate the risk of colostrum containing antimicrobial residues above the MSC values , as found in our study, is low, but further research on the risk of low levels of antimicrobial residues on the selection of resistant bacteria in the neonatal calf is warranted, however falling beyond the scope of this study.

### Role of administration route on AMR selection

Generally, it is assumed that orally and parenterally applied antimicrobials (i.e. systemic AMU) have a greater impact on the selection and spread of AMR, opposed to locally applied AMs (e.g. intramammary use)^10^. When systemic AMU is compared to local use, different microbial populations located in different body sites are exposed, therewith increasing the risk to select for AMR. Currently, in veterinary medicine, the first line use of 1st generation cephalosporins is discouraged as they are classified as Highly Important Antimicrobials by the WHO and as ‘caution’ by the European Medicines Authority. However, the local (i.e. intramammary) use of these antimicrobials might pose less risk compared to parenteral treatments. Prudent antimicrobial use guidelines do not consider the administration route. Future (in vivo) research is needed to better understand how different administration routes may affect the emergence and spread of antimicrobial resistance. To combat AMR, overall responsible AMU is advocated: administration of the right drug, in the right dose, in the right way and with the right frequency and duration. Where possible, AMU should be guided by antimicrobial susceptibility testing. Whenever alternatives to substitute antimicrobials are available, these should be applied. The use of selective antimicrobial dry cow and mastitis treatments in this respect have shown to be beneficial in promoting judicious use of antimicrobials in the treatment of intramammary infections in dairy cows^45,46^.

## Conclusion

In our study we found no evidence supporting selection of ESBL-producing *E. coli* in bovine feces or in manure slurry after intramammary use of commercial CP or CL containing products, using laboratory competition assays based on existing literature on pharmacodynamics and pharmacokinetics. The calculated maximum concentrations of CP and CL in the bovine gut and in manure slurry after intramammary administration will be substantially lower than the found minimum selective concentrations of CP and CL found to select for ESBL-producing *E. coli* in these environments. These results suggest that intramammary use of 1st generation cephalosporins in dairy cows do not contribute for the selection of ESBL producing bacteria, however confirmation with field studies are of paramount importance in the near future.

## Materials and methods

### Literature search

In November and December 2019, a non-systematic scientific literature search was conducted. Besides, a search was performed in publicly available scientific reports from authorizing institutes (i.e. European Medicines Agency) and monographs from veterinary pharmacology organizations, to gather information on pharmacokinetics and excretion routes of CP and CL after intramammary application in dairy cows. Scientific literature was searched for using Pubmed Central and Google Scholar using search strings “cephapirin”, “cefalonium”, “intramammary”, “dairy” and “pharmacokinetics”. Additionally, scientific information was searched for on usual intramammary treatment incidences and on feces and manure slurry production of dairy cows. Based on these findings, calculations were made to estimate the maximum concentrations of CP and CL in the bovine GIT and in manure slurry.

### Pharmacokinetics of cephapirin and cefalonium

CP is used in dairy cows for treatment of intramammary infections both at drying off and during lactation^18,22,47^. Most of the intramammary administered CP is excreted via milk, although a substantial part of CP can cross the milk-blood barrier and is absorbed systemically^15,19^. CP is largely metabolized in the udder, liver, and kidneys to desacetyl-cephapirin which still has bactericidal activity, although it is less potent compared to its parent drug^26–28^. Systemically absorbed CP is mainly eliminated by the urinary route and to a smaller extent by the biliary route and low concentrations of the parent drug or its metabolite can therewith enter the gut lumen^15,18,48^. Like CP, CL will be mainly systemically absorbed after intramammary application however, it is not metabolized and like most cephalosporins, it is eliminated from the body largely via urine after renal tubular secretion and/or glomerular filtration^22,23^. The assumptions made for calculating the presumptive concentrations of CP and CL in the bovine gut and in slurry can be found in Table 1. The background information for these assumptions can be found the supplementary file^22,23^.

Daily production of manure in dairy cattle is relatively variable and is amongst others dependent on daily milk yield and dry matter intake^20,21,49^. Based on estimates on daily manure production and daily urine production, it can be assumed that the daily feces production will be 40–50 kg/day for lactating dairy cows and around 25 kg/day for dry cows.

### Competition assays

Institutions from four different European countries (the United Kingdom, Quality Milk Management Services; Germany, University of Applied Sciences; Belgium, Animal Health Care Flanders; the Netherlands, Utrecht University) were participating in the study. Between March and June 2019, three ESBL producing *E. coli* isolates and three non-ESBL producing *E. coli* isolates were selected isolated from (bulk) milk, feces or bedding from three different dairy farms per country and sent to the laboratory of Utrecht University, The Netherlands. After retrieval, confirmatory analyses were performed. *E. coli* isolates able to grow on MacConkey agar plates containing 0.25 µg/ml CTX (the ECOFF for CTX) were considered as ESBL-producers (mic.eucast.org/search/). For each country, three sets of competition assays to study the potential effects of intramammary applied CP and CL to select for ESBL *E. coli*-producers were performed; in enriched culture medium (experiment 1), fecal fermentations (experiment 2) and dairy manure slurry (experiment 3).

### Experiment 1 in enriched medium

Per country, one colony from each of the three ESBL and three non-ESBL *E. coli* producer strains were dispensed in tubes containing 3 ml Lysogeny Broth (LB) medium (Oxoid, Tritium, The Netherlands) and incubated overnight in a shaking incubator at 37 °C. The next day, the ESBL and non-ESBL overnight cultures were mixed in a 1:3 ratio. Three µl of this mixture was added to nine different tubes containing 3 ml of LB medium. Additionally, antibiotics were added to seven of the nine tubes (0.08, 0.8 and 8.0 µg/ml of CP; 0.04, 0.4 and 4.0 µg/ml of CL; 0.25 µg/ml CTX). Two tubes containing no antibiotics were used as negative controls.

Except for the tubes with no antibiotics, diluted and plated immediately (baseline, t = 0), all the other tubes were incubated for six hours in a shaking incubator at 37 °C. After six hours, a 1:10 dilution in LB medium was prepared of each sample. Each dilution was subsequently inoculated on five MacConkey agar plates using a 10 µl loop and incubated overnight at 37 °C. After overnight incubation, the five plates per sample were used to select 96 individual colonies, which were phenotypically determined as *E. coli* based on their morphology and pink colour due to lactose fermentation. These colonies were subsequently added to a 96 wells plate containing 100 µl of LB.

The suspensions from the 96 wells plate were then transferred (without incubation) to two different square MacConkey agar plates (per sample) (MacConkey and MacConkey + 0.25 µg/ml CTX) using 96 tip stamps that absorb the bacterial suspension and dispense it on the square plates by contact. After overnight incubation, the stamped MacConkey plates were used to assess the percentage of CTX resistance present in each of the conditions.

### Experiment 2 in fecal fermentations

Fresh fecal samples collected from 10 different healthy cows from three different farms (at least three cows per farm) from each of the four countries were transported under refrigeration (2–8 °C) to the laboratory of Utrecht University and arrived within 24 h after sampling. Per country, fecal samples were mixed to create one pooled fecal sample. Thereafter, 0.1 g of this pooled sample was added to nine 1.5 ml screwcap micro centrifuge tubes. Overnight cultures of the ESBL and non-ESBL *E. coli* strains (*E. coli* strains per country of origin) on blood agar plates were used to make a 0.5 McFarland suspension in saline for each individual strain. Equal parts of these McFarland suspensions from both the ESBL and non-ESBL *E. coli* strains were mixed in a fresh tube creating an 1:1 ESBL and non-ESBL mixture. This mixture was used to spike the nine fecal samples (per individual country) with ± 10^7^ CFU/gram. This concentration of 10^7^ CFU *E. coli*/gram feces was chosen because previous research has shown that feces contain between 6.55 × 10^6^ and 7.60 × 10^6^ CFU/gram dry matter^50^. Therefore, choosing 10^7^ CFU/gram for spiking ensures that the ESBL and non-ESBL mixture is present at a higher level than the original *E. coli* in the sample, while guaranteeing that sufficient growth remains possible. These nine spiked fecal samples were then placed in an anaerobic hood where, under anaerobic conditions, 900 µl of anaerobic Standard Ileal Efflux Medium (SIEM,) was added to each tube^51^. Additionally, antibiotics were added to seven of the nine tubes (i.e. 0.08, 0.8 and 8 µg/ml of CP and 0.04, 0.4 and 4 µg/ml CL and 0.25 µg/ml CTX). The two remaining tubes contained no antibiotics (baseline, t = 0 h and blank, t = overnight). Immediately after the addition of the SIEM medium, the t = 0 h samples were taken from the anaerobic hood and plated onto five MacConkey agar plates. These plates were incubated overnight at 37 °C. The other eight tubes were incubated overnight at 37 °C and 300 RPM in the anaerobic hood on a shaking heating block. After overnight incubation, these eight tubes were each plated onto five MacConkey agar plates using a 10 µl loop. These plates were subsequently incubated overnight at 37 °C. After overnight incubation, the same procedure as in experiment 1 was followed to inoculate four different square MacConkey agar plates (per sample) (MacConkey and MacConkey + 0.25 µg/ml CTX). The tubes containing the leftovers from the fecal fermentations were centrifuged at maximum speed for one minute, after which the supernatant was transferred to a fresh 1.5 ml micro centrifuge tube and stored at − 20 °C for DNA isolation. After overnight incubation, the stamped MacConkey plates, were used to assess the percentage of CTX resistant colonies present in each of the conditions.

### Experiment 3 in manure slurry fermentations

Per country, from one randomly chosen dairy farm, a sample from the manure pit was taken. This sample was cooled and transported to the laboratory of Utrecht University within 24 h. The same procedure as for the pooled fecal samples in experiment 2 was followed, except for the temperature, that was kept at 17 °C. The circumstances in a manure slurry pit are different compared to the circumstances in the intestinal lumen. The temperature is much lower, around 15 °C, maximum in summer around 18–19 °C, under Western European conditions, and it contains both urine and feces, wastewater and sometimes waste milk^52^.

### Metagenomic sequencing

To investigate the selection on ESBL production, DNA was isolated from 1 ml of feces and 1 ml of manure slurry of all nine tubes after experiment 2 and 3, using the protocol as described for the EFFORT-project^53^. Illumina sequencing was performed as described by the manufacturer using 150 bp paired end Illumina Novaseq sequencing Libraries were created using the Ilumina Nextera XT DNA Library Preparation Kit according to the manufacturer’s protocol^54^. Resulting DNA reads were processed using FastDeME according to the default settings^55^. Reads were processed using FastP to remove poor quality reads, sequencing adapters and barcodes^56^. To investigate the resistome, Kaiju was used to profile the reads for bacterial species identification and the tool KMA was used to detect allignments to known antimicrobial resistance genes with the default Resfinder database^57,58^. The sequence depth of each resistance gene was adjusted for sequencing depth to depth per gigabase. Resistance classes were obtained from the Resfinder database^59^ and hits to each resistance gene were summed per antimicrobial class. Sequence data is available under accession PRJEB49007 at the sequence read archive (SRA). Results were visualized using Microsoft Excel.

## Supplementary Information

Supplementary Information.
