# Supplementary material for: Potential of ESBL-producing Escherichia coli selection in bovine feces after intramammary administration of first generation cephalosporins using in vitro experiments
Source: Sci Rep. 2022 Sep 5;12:15083. doi: 10.1038/s41598-022-15558-z (PMC9445091; doi:10.1038/s41598-022-15558-z)
Supplement: Supplementary file 1 — Supplementary Information. [file 41598_2022_15558_MOESM1_ESM.docx]

**Pharmacokinetics of cephapirin and cefalonium, a literature review.**

CP is used in dairy cows for treatment of intramammary infections both at drying off and during lactation. The commercially available products containing benzathine and sodium salts of CP are applied in doses of 200-300mg/quarter, either applied at once in all quarters at drying off, or during lactation applied in one quarter four times with an interval of 12 hours as treatment ^1^. The dihydrate of CL is used in commercial products at drying off in recommended doses of 250 mg/quarter ^2, 3^.

Most of the intramammary administered CP is excreted via milk, although a substantial part of CP can cross the milk-blood barrier and is absorbed systemically. Based on animal experiments, systemic absorption will however not be more than 41% of the initially applied dose ^4, 5^. CP is largely metabolized in the udder, liver, and kidneys to desacetyl-cephapirin which still has bactericidal activity, although it is less potent compared to its parent drug ^6-8^. Systemically absorbed CP is mainly eliminated by the urinary route and to a smaller extent by the biliary route and low concentrations of the parent drug or its metabolite can therewith enter the gut lumen ^1, 5, 9^.

Ray et al., found peak concentrations in fresh rectally derived bovine feces of 2.04 ng/kg and 2.12 ng/kg, respectively, four and six hours after a single intramammary application of 1.200 mg CP (300 mg/quarter) ^5^. After eight hours, CP was below the detection limit in feces. In urine, the concentrations of CP were 133 and 480 µg/L after four and eight hours, respectively, and declined thereafter. Intravenous administration of 8,6 mg/kg sodium cephapirin in adult dairy cows (equals 5,160 mg CP for a cow of 600 kg bodyweight) resulted in a peak bile concentration of 10.3 µg/g six hours after administration ^1^. Intramammary applied CP and its metabolites can be detected in milk samples up to 48 hours after the last intramammary administration. A substantial part of CP and its metabolites will however be degraded before it is excreted in the milk with estimated half-lives of respectively three and eight hours ^10^.

Like CP, CL will be mainly systemically absorbed after intramammary application however, it is not metabolized and like most cephalosporins, it is eliminated from the body largely via urine after renal tubular secretion and/or glomerular filtration ^3, 11^. Although CL is relatively insoluble, an experiment with radiolabelled CL indicated that over 50% of the initial intramammary applied dose was systemically absorbed over a 21 days period. After a single intramammary treatment with 250 mg CL in each quarter of adult dairy cows, peak serum concentrations of 0.42 µg/ml were found eight hours after administration. In this experiment, 29% and 2% of the initial dose was excreted via urine and feces, respectively, during the first three days after treatment. In the urine, peak concentrations of 24.1 µg/ml were found 12 hours after administration which gradually decreased in the days thereafter. Another experiment showed that 40% of intramammary-infused radiolabelled CL was excreted in the first seven days after administration: 33-36% in the urine and 3-5% in the feces ^3, 11^.

Daily production of manure in dairy cattle is relatively variable and is amongst others dependent on daily milk yield and dry matter intake ^12-14^. Based on estimates on daily manure production and daily urine production, it can be assumed that the daily feces production will be 40-50 kg/day for lactating dairy cows and around 25 kg/day for dry cows.

**References**

1. The European agency for the evaluation of medicinal products, veterinary medicines and information technology. Cefapirin Summary Report 2. (2001).

2. Bryan, M. A., Heuer, C. & Emslie, F. R. The comparative efficacy of two long-acting dry-cow cephalonium products in curing and preventing intramammary infections. *N. Z. Vet. J.* **59**, 166-173 (2011).

3. European Medicines Authority Committee for Veterinary Medicinal Products. Cefalonium Summary Report 2. *European Medicines Authority* (2002).

4. Stockler, R. M., Morin, D. E., Lantz, R. K. & Constable, P. D. Effect of milking frequency and dosing interval on the pharmacokinetics of cephapirin after intramammary infusion in lactating dairy cows. *J. Dairy Sci.* **92**, 4262-4275 (2009).

5. Ray, P., Knowlton, K. F., Shang, C. & Xia, K. Development and validation of a UPLC-MS/MS method to monitor cephapirin excretion in dairy cows following intramammary infusion. *PloS one* **9**, e112343 (2014).

6. Moats, W. A., Anderson, K. L., Rushing, J. E. & Buckley, S. Conversion of cephapirin to deacetylcephapirin in milk and tissues of treated animals. *J. Agric. Food Chem.* **48**, 498-502 (2000).

7. Cortinhas, C. S., Oliveira, L., Hulland, C. A., Santos, M. V. & Ruegg, P. L. Minimum inhibitory concentrations of cephalosporin compounds and their active metabolites for selected mastitis pathogens. *Am. J. Vet. Res.* **74**, 683-690 (2013).

8. Jones, R. N. & Packer, R. R. Cefotaxime, cephalothin, and cephapirin: antimicrobial activity and synergy studies of cephalosporins with significant in vivo desacetyl metabolite concentrations. *Diagn. Microbiol. Infect. Dis.* **2**, 65-68 (1984).

9. AAVPT, American Academy of Veterinary Pharmacology and Therapeutics. Veterinary Clinical Drug Information Monographs. Cephapirin. (2007).

10. Cagnardi, P. *et al*. Pharmacokinetics in foremilk and antimicrobial activity of cephapirin following intramammary administration in healthy and Staphylococcus aureus-infected cows. *N. Z. Vet. J.* **62**, 146-151 (2014).

11. European Medicines Authority Committee for Veterinary Medicinal Products. Cefalonium Summary Report 1 *European Medicines Authority* (1999).

12. Nennich, T. D. *et al*. Prediction of manure and nutrient excretion from dairy cattle. *J. Dairy Sci.* **88**, 3721-3733 (2005).

13. Mgbeahuruike, A. C., Nørgaard, P., Eriksson, T., Nordqvist, M. & Nadeau, E. Faecal characteristics and milk production of dairy cows in early-lactation fed diets differing in forage types in commercial herds. *Acta Agriculturae Scandinavica, Section A—Animal Science* **66**, 8-16 (2016).

14. Lorimor, J. & Powers, W. Manure Characteristics. Manure Management Systems Series. **MWPS-18 Section 1** (2004).

stylefix
